# Banking Umbilical Cord Blood (UCB) Stem Cells: Awareness, Attitude and Expectations of Potential Donors from One of the Largest Potential Repository (India)

**DOI:** 10.1371/journal.pone.0155782

**Published:** 2016-05-26

**Authors:** Deeksha Pandey, Simar Kaur, Asha Kamath

**Affiliations:** 1 Department of Obstetrics & Gynaecology, KMC Manipal, Manipal University, Karnataka, India; 2 Department of Community Medicine, KMC Manipal, Manipal University, Karnataka, India; Universidade de São Paulo, BRAZIL

## Abstract

**Background:**

The concept of Umbilical Cord blood (UCB) stem cells is emerging as a non-invasive, efficacious alternative source of hematopoietic stem cells to treat a variety of blood and bone marrow diseases, blood cancers, metabolic disorders and immune deficiencies. Aim of the present study was to determine the level of awareness about banking UCB among pregnant women in India. We also assessed patient perception for banking of UCB and explored the patient expectations of banking UCB in future. This is the first study to assess current attitudes, in a sample population of potential donors from one of the largest potential UCB repository (India). Obtaining this information may help optimize recruitment efforts and improve patient education.

**Material and Method:**

Present explorative questionnaire based survey included 254 pregnant women in the final analysis.

**Results:**

We established only 26.5% pregnant women in our study population knew what exactly is meant by UCB. A large proportion (55.1%) was undecided on whether they want to bank UCB or not. Women were more aware of the more advertised private cord blood banking compared to public banking. More than half of the pregnant women expected their obstetrician to inform them regarding UCB. One-third of the women in our population had undue expectations from banking of the UCB.

**Conclusion:**

Obstetricians should play a more active role in explaining the patients regarding pros and cons of UCB banking.

## Introduction

As the newborn is delivered, and the umbilical cord is divided, blood can be collected from the segment of cord still attached to the placenta. Stem cells retrieved from the blood in the remaining segment of the umbilical cord and placenta are known as ‘umbilical cord blood (UCB) stem cells’. This blood which is of no use to the mother or the baby and has been treated as a medical waste for centuries is a rich source of stem cells. UCB stem cells are unique and have many promising uses for the future. As these cells are naïve, on allogeneic transplantation, they produce an attenuated donor-derived immune response and thus have a lower incidence of graft-versus-host reaction when compared to other sources of stem cells (bone marrow or peripheral cells). Unlike other sources, these can also be transplanted even without an identical HLA match. The collection procedure is easy and without any risk to the donor (mother or baby). [1] Unlike embryonic stem cells these are not ethically controversial. Despite many advantages, UCB stem cells have certain limitations. It requires appropriate collection, processing, and storage and a single UCB stem cell unit has remained a limiting factor for stem cell transplant in adult recipients. [2] Likewise, a patient’s own UCB may not be useful in many cases as the precursor of the disease may be in the UCB stem cells as well.

UCB can be banked in the following two ways: 1) Private UCB banks–wherein the UCB of a newborn is stored at a certain cost. These UCB can be used only by the child or his family if a need arises; 2) Public UCB banks–these are similar to blood-banks. Here any pregnant woman can enrol to donate UCB at the time of child birth free of cost, and anyone in need can utilize it at a certain cost.

In reality, private UCB banking is advocated only in a few cases, such as when parents of the newborn are known carriers of mutations associated with genetic diseases of the blood, immune system or inborn errors of metabolism. Private banking is also indicated when there is an older affected sibling with one of these diseases who would be a candidate for UCB stem cell transplantation.[3]

However, because parents are made more aware of private blood banks through advertisements and media coverage, in our day to day practice we observed that parents appeared to be more steered towards private vs public storage of their infant’s cord blood. [4] From these sources, they are often misinformed about the unrealistic future usage, for commercial banks often list many conditions that might be treated in the future by as yet undeveloped stem cell therapies for regenerative medicine. [5] Experts do no recommend UCB banking for unproven future uses. [3,6]

Surveys done in Europe and Canada had revealed that pregnant women have very limited knowledge about UCB and its banking. [7,8] The aim of this study was to assess the awareness, attitude and expectations of UCB banking among pregnant women in India. This information derived from this sample population of potential donors from one of the large potential UCB repository (India) may aid in the development of better recruitment strategies by public and private banks, and better practice guidelines for clinicians.

## Material and Methods

An explorative questionnaire based survey was used, following the ethical guidelines mandated by the Institutional Review Board. The project protocol was approved by Ethics Committee, Kasturba Hospital Manipal, Karnataka, India (IEC 382/2013). Pregnant women attending the antenatal care clinic in our hospital were recruited for the study. The only criteria for exclusion was the unwillingness of a woman to participate in the study. Women (one to four at a time) were introduced to the study by one of the investigators and were encouraged to participate in the study. Participants were provided with a written summary of information about the study and were allowed to ask questions. Written consent for participation was obtained. The consent forms and the filled questionnaire were collected separately by two different investigators to avoid personal identification. Thus, anonymity and confidentiality of the participants were guaranteed. The personal right to withdraw from the survey at any moment was ensured.

The study was conducted in two steps. Step 1) Women were asked to answer the questionnaire having 15 items (5 for awareness, 5 for attitude and 5 related to their expectations from UCB). Step 2). The investigators provided answers to educate the participants to make them aware of the myths and realities of UCB banking. Later on, responses to the questionnaire were collated by the investigators and were analysed.

### Survey instrument

Questionnaire (Data in S1 Text)–A questionnaire having 15 questions was prepared. The first five questions (question number 1–5) were designed to determine the baseline awareness of women about UCB banking. The next five (question number 6–10) questions were to ascertain the attitude of these women towards UCB banking. The last five questions (question number 11–15) were designed to explore the expectations of the participants about the banking of UCB. The questionnaire was developed in English. For construction and content validity, it was reviewed by five independent experts (one each from five related specialities: Obstetrics & Gynaecology, Transfusion Medicine, Paediatrics, Nursing, and Community Medicine). There was 80% or more agreement for 12 out of 15 questions and their wording, which were left as is. For the other three questions, suggested changes were made. This English questionnaire was then translated into Kannada, which is the local language of our target population. Two experts in the language then validated it for language appropriateness. The questionnaire was tested for face validity in a pilot study, with ten pregnant women from our antenatal clinic to ascertain that the questions were acceptable and the wording was well understood by the respondents. ***(Kindly refer to S1 Text).

Sample size calculation: To estimate the level of awareness regarding UCB banking among antenatal patients, at 66% with a relative precision of 10% for a confidence level of 95%, a sample size of 198 was calculated. Considering, the possibility of an incomplete response, 280 women were required to be included in the study.

### Statistical Analysis

Data obtained were analysed using SPSS statistical software version 16. The responses of the participants to questions were analysed according to the stratification. Chi-Square test was used to assess the significance of the responses and a P value < 0.05 was considered statistically significant.

## Results

A total of 300 women were recruited for the study, and out of these 13 refused to participate. Two hundred and eighty seven (287) women filled the given questionnaire. However 33 questionnaires were found to be incomplete, and thus had to be excluded, bringing the number of women included in the final analysis to 254. Out of these 145 (57.1%) were primigravida. Most of the women (147; 57.9%) in the study population were in their third trimester. Around 68% of women were in the age group 20–30 years. Regarding educational status 36.2% had graduate level education, 51.1% had completed their secondary education, and 12.6% had finished primary education. None of the women were illiterate (Table 1).

**Table 1:** Demographic characteristics of the population studied.

Characteristics	Number of Participants (N = 254)	Percent (%)
**Parity**		
Primi	145	57.1
Multi	109	42.9
**Trimester**		
1^st^ trimester	32	12.6
2^nd^ trimester	75	29.5
3^rd^ trimester	147	57.9
**Age**		
< 20 years	16	6.3
20–30 years	173	68.1
> 30 years	65	25.6
**Educational Status**		
Primary	32	12.6
Secondary	130	51.1
Graduation	92	36.2

### A. Awareness regarding UCB banking

Overall awareness of the general understanding of banking was poor in the study population. Only 26.5% women knew exactly what ‘umbilical cord blood (UCB) stem cell banking’ meant. 31% knew about ‘private cord blood banking’ while only 16% were aware of the concept of ‘public cord blood banking’. Less than one quarter (22.4%) of the women had the correct information on the primary indications of cord blood banking. Only 18.1% of the women knew that the likelihood of needing to use stored UCB for self or family members in the future is very low (1 in 2700). (Table 2)

**Table 2:** Awareness regarding stem cell banking.

Questions on Awareness(1–5)	Correct Response	Participants N = 254 (% Correct)
**1. Cord Blood stem cell Banking**	Banking of blood from the placental side of the cord	**68 (26.5%)**
**2. Public Cord Blood Bank**	Where anyone can donate (free of cost); anyone in need can take (at some cost)	**41 (16.1%)**
**3. Private Cord Blood Bank**	Where anyone can donate (at some cost); only his family can take (free of cost)	**79 (31.1%)**
**4. Indication for banking cord blood stem cells**	Family history of metabolic and blood disorders/older child having these problems	**57 (22.4%)**
**5. Likelihood of using the stored cord blood stem cells in future**	If 2500 have stored one might need it	**46 (18.1%)**

We then correlated the level of education with the level of awareness. For all the questions asked related to the overall awareness of cord blood banking, the results showed high dependence on the level of education. (Table 3)

**Table 3:** Relationship between awareness levels and educational status.

Questions (1 to 5)	Primary n = 32 (%)	Secondary n = 130 (%)	Graduation n = 92 (%)	Statistical significance (P value) Chi Square
**1.Cord Blood stem cell Banking (n = 68)**	3 (9%)	28 (21.5)	32 (34.7)	0.002
**2.Public Cord Blood Bank (n = 41)**	2(6%)	15 (11.5)	21 (22.8)	0.039
**3.Private Cord Blood Bank (n = 79)**	**3 (9%)**	**39 (30)**	**30 (32.6)**	**0.001**
**4.Indication for banking cord blood stem cells (n = 57)**	1 (3%)	25 (19.2)	27 (29.3)	0.003
**5.Likelihood of using the stored cord blood stem cells in future (n = 46)**	3(9%)	18 (13.8)	20 (21.7)	0.071

### B. Attitude towards UCB banking

**Fig 1:**
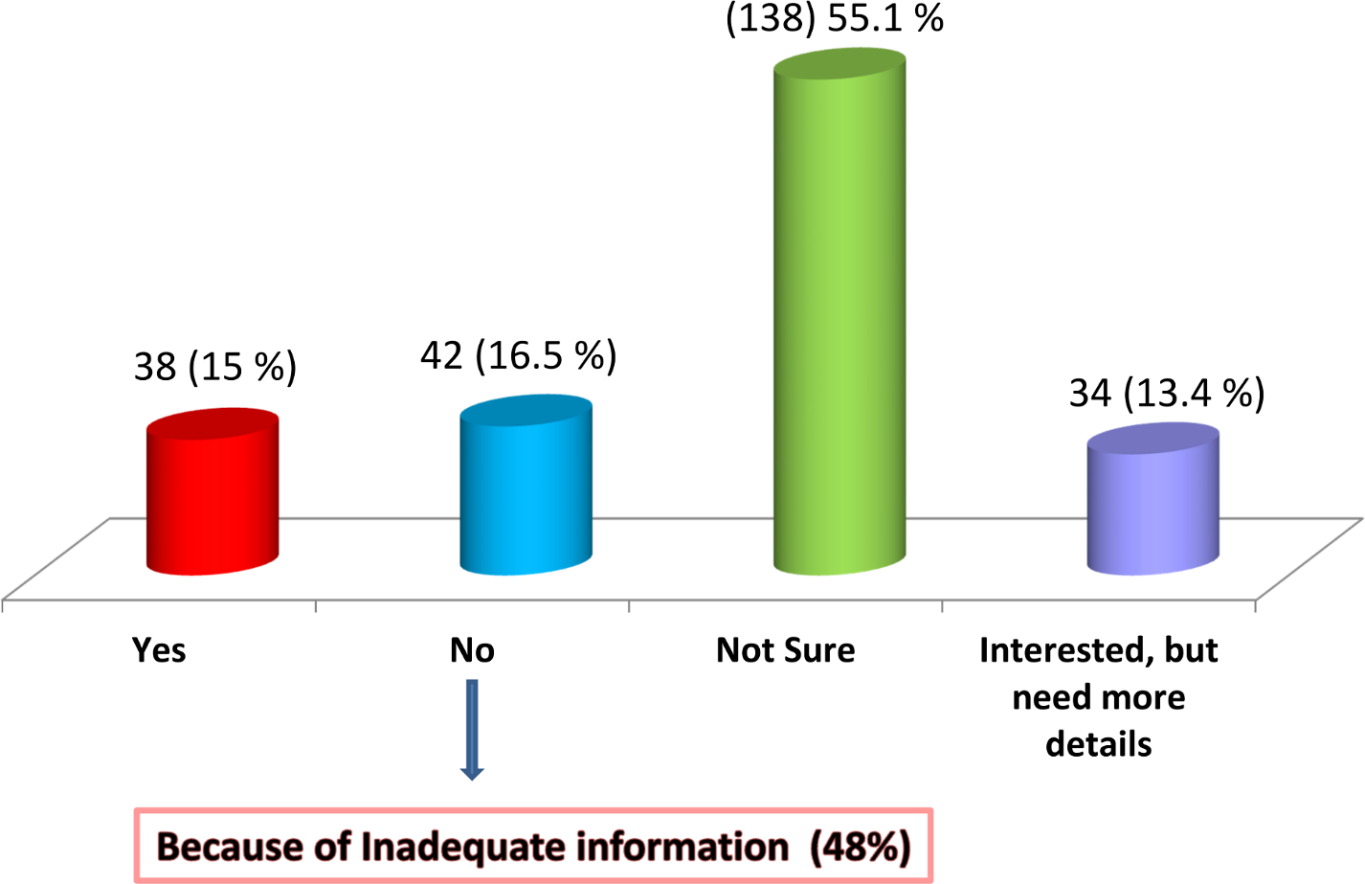
Willingness to store cord blood stem cells for the index pregnancy.

Only 15% of the women showed willingness for CBSC banking at the time of their delivery. Another 13.4% of women were interested, but needed further details on the process before deciding. A large proportion (55.1%) was unsure of whether they wanted UCB or not at the time of the study. (Fig 1)

Among those who did not want UCB banking (16.5%), more than half of them (62%) were against it because they felt they did not have enough information about it. The remaining 38% believed it is costly and could not afford it. Out of those 15% who wanted UCB banking; 49% wanted to bank it in a private bank, and 19% wanted it in a public bank. The remaining 32% did not know the difference between the two banking systems.

**Fig 2:**
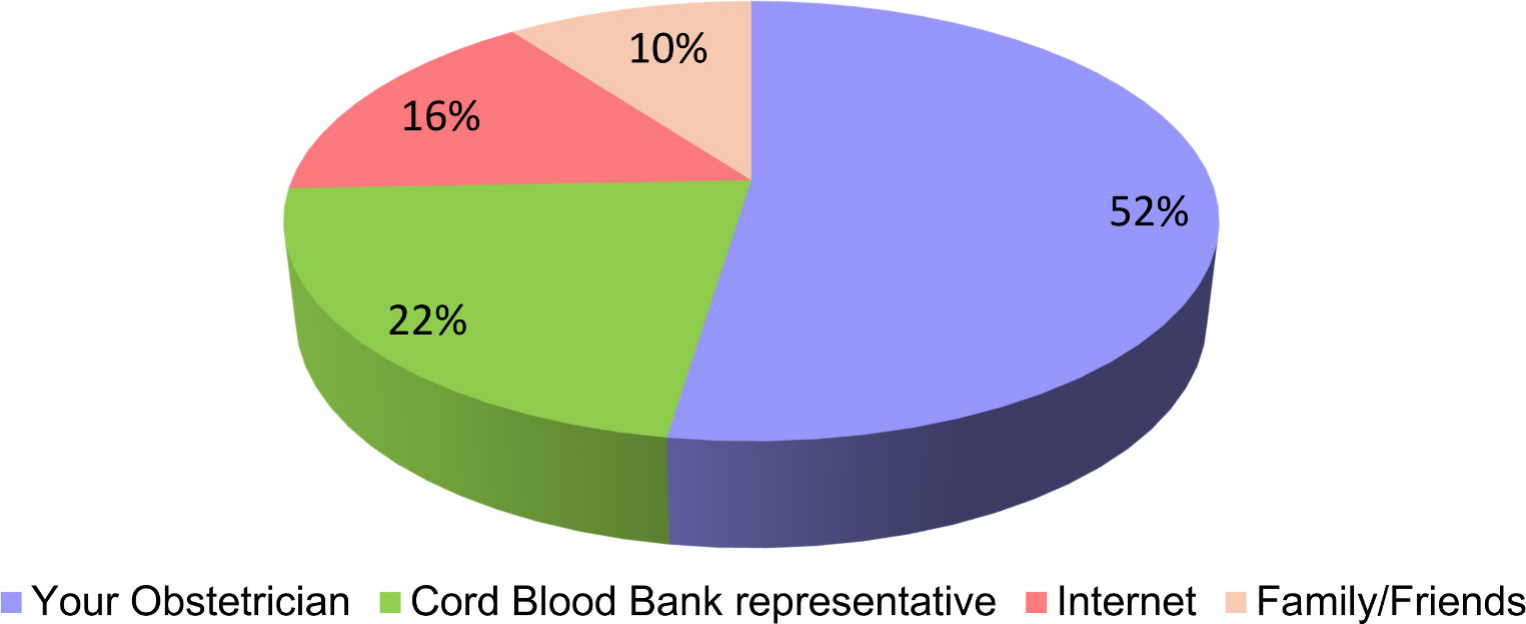
Preferred source to gather information related to the issues of cord blood banking.

When asked from where or whom they would prefer to get the information on this issue, 52% wanted it from their obstetricians, 22% from UCB representative, 16% from the internet and 10% from family/friends. (Fig 2)

**Fig 3:**
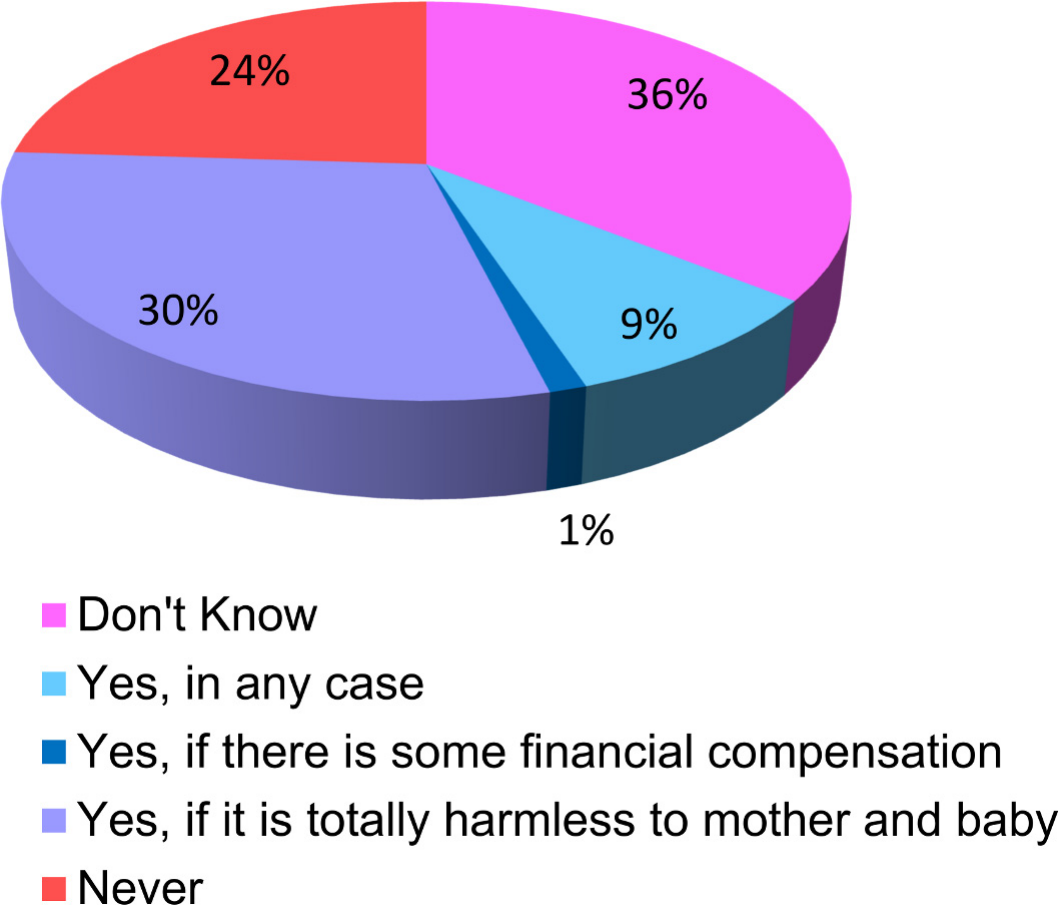
Willingness to bank cord blood for research (if given an option), keeping in mind the larger picture of future benefit to the society.

When approached on if they would like to bank cord blood for research (if given an option), keeping in mind the larger picture of future benefit to the society, the responses were diverse. Approximately one third (30%) said they are ready to store it for research purpose if it is totally harmless to the mother and the baby. Only 1% said they will donate it if there is some financial compensation. While 9% were willing to do it irrespective of any condition 24% did not want it at any cost. A big proportion of women (36%) were still indecisive on this matter. (Fig 3)

### C. Expectations from UCB banking

In our study population 33% of women were unsure what to expect out of UCB banking. Around one-fifth (18%) thought it would be useful for chronic diseases such as hypertension and diabetes. Seven percent thought it would be useful to treat cancer. Five percent believed it to be useful for both chronic illness and cancers. 13% felt it would be useful in regenerating organs in future. One-fourth (24%) of these women were over optimistic, and they felt that it could be used for any of the indication above.

## Discussion

In the present study, we established that the overall awareness regarding UCB banking was poor among pregnant women. Women were more aware of the much hyped private cord blood banking compared to public banking. More than half of the pregnant women expected their obstetrician to inform them regarding UCB. One-third of the women in our population had unrealistic expectations from banking of the UCB. This is the first study from India (a region of a huge potential repository of diverse public UCB banks) exploring the awareness and expectation of potential donors for UCB banking (pregnant women).

Only 26.5% pregnant women in our study population knew exactly what is meant by UCB. Likewise poor or a very poor awareness was seen in other studies from Canada and Europe. [8,9] Overall awareness of cord blood banking, showed high dependence on the level of education. Similar trend was seen in Chinese population in a recent study. [10]

In a multicentre study involving five European nations (France, Germany, Italy, Spain, and the UK) found a strong preference for public banking (76.5%) among the pregnant women. [9] In our study population 31% knew about ‘private UCB banking’ while only 16% were aware of the concept of ‘public UCB banking’. A Public Bank is the area that is being promoted to expand more ethnically diverse inventory of UCB units. [11] In India, the first public UCB repository became functional in early 2000. [12] However since then time has not advocated expected progress in this field of utmost public importance.

We found a mixed reaction for donating UCB for research when it is not suitable for transplantation. We believe this was due to the overall unawareness of this issue. In Europe, almost all of pregnant women (91.8%) who wish to store their child’s cord blood were willing to donate it to research where the awareness level was much higher among the participants. [9]

Obstetricians and others health care professionals delivering antenatal care should take it as a primary responsibility to increase awareness of umbilical cord blood donation to develop and expand public banking activities.[11] In a study of awareness and acceptance of UCB in the United States revealed that 80% of the obstetricians felt confident discussing UCB options with their patients; however, 49% indicated that they have insufficient knowledge of cord blood donation to answer effectively patients’ questions about donation.[13] A recent study from India who surveyed a group of general population and doctors, and found out that 58% of the doctors and 82% of the lay persons were either unaware or misinformed about the indications of UCB transplant. Despite this huge lack of knowledge 40% of doctors and 69% of the participants from the general population were wiling for UCB banking for their child. The study concluded that the obstetricians and the paediatricians should take a central role in providing the correct information to would be parents to help them in taking a correct decision. [14]

Currently private banking of UCB is not recommended for unidentified possible future use.[4] The American College of Obstetricians and Gynaecologists (ACOG), American Academy of Paediatrics (ACP) and American Society of Bone Marrow Transplant (ASBMT) also do not advocate private storage unless there is an identified need in the family in which banked cord blood would offer a benefit.[1,11,15,16] The ACOG committee emphasises balanced and accurate information should be provided to the patients with regards to the advantages and disadvantages of public versus private banking.[17] The remote chance of an autologous unit of umbilical cord blood (as in a private bank) being used for a child or a family member (approximately 1 in 2,700 individuals) should also be disclosed clearly. [11,15] Though it has to be kept in mind that this statistics is based on the use of cord blood only as a source of hematopoietic stem cells and it also does not highlight the importance of family banking in the setting where one child has a transplantable disease.

There is less awareness with regards to the Public banks among the population. It is rationale to consider that lack of availability of a public bank in the community contributes for the lack of awareness. Furthermore as stated in a recent study nearly 90% of existing public cord blood banks have declared that they are struggling to maintain their financial sustainability.[18] Increasing awareness of existence of such banks to the potential donors and potential recipients will be the first step to increase its utilization which will help these banks to sustain and more banks to establish in future.

Obstetricians should assume that pregnant women are poorly informed about cord blood banking. [7] One of the goals of antenatal care should be to ensure that every pregnant woman has an opportunity to make a well-informed decision about cord blood banking.[7] The common observation is the commercial (private) banks get involved in a direct-to-consumer advertising campaigns often bypassing obstetricians and other health care providers. This is a prime concern that has led to more misconceptions in our society. Active involvement by obstetricians would allow patients to be educated unbiased with more evidence-based scientific evidence to reach a greater number of potential donors without significantly increasing demands on the public banking program.

## Conclusion

Overall awareness regarding UCB banking was poor among pregnant women in India. Women were more aware of the more aggressively marketed private cord blood banking compared to public banking. Obstetricians should play a more active role in explaining the patients regarding pros and cons of stem cell banking.

## Supporting Information

S1 Table We confirm that the data set that we have uploaded as S1 Table constitute the minimal dataset underlying the findings of our paper. (XLSX)

S1 Text Q stem cells. This is the survey instrument used in our study.
(DOCX)
