# Supplementary material for: Banking Umbilical Cord Blood (UCB) Stem Cells: Awareness, Attitude and Expectations of Potential Donors from One of the Largest Potential Repository (India)
Source: PLoS One. 2016 May 26;11(5):e0155782. doi: 10.1371/journal.pone.0155782 (PMC4881935; doi:10.1371/journal.pone.0155782)
Supplement: S1 Text — This is the survey instrument used in our study. (DOCX) [file pone.0155782.s002.docx]

**Banking cord blood stem cells: awareness, attitude and expectations among pregnant women in India**

**Study No**

**Hospital number (as reference):**

**Phone number (as reference):**

**Age:**

**Parity:**

**POG:**

**Education:**

**Occupation:**

**Per capita income:**

***Awareness (1-5)***

1. What is cord blood stem cell banking?
2. Banking of a mother’s blood during delivery
3. Banking of a newborn’s blood in blood bank
4. Banking of blood from umbilical cord attached to the baby
5. Banking of blood from the placental side of the cord
6. What are ‘Public cord blood banks’?
7. Where anyone can donate (free of cost); anyone in need can take (free of cost)
8. Where anyone can donate (at some cost); anyone in need can take (free of cost)
9. Where anyone can donate (free of cost); anyone in need can take (at some cost)
10. Where anyone can donate (at some cost); anyone in need can take (at some cost)
11. What are ‘Private cord blood banks’?
12. Where anyone can donate (at some cost); anyone in need can take (at some cost)
13. Where anyone can donate (at some cost); only his family can take (free of cost)
14. Where anyone can donate (at some cost); anyone in need can take (free of cost)
15. Where anyone can donate (at some cost); only his family can take (at some cost)
16. Who should bank cord blood stem cells for the sake of their own family
17. Family history of diabetes and high blood pressure
18. Family history of metabolic and blood disorders/older child having these problems
19. All those who can afford
20. All who want to secure health of their family
21. What do you think is the likelihood of you (or your family member) using the cord blood stem cells you have stored
22. Everyone might need it at some point of time
23. If 25 have stored 1 might need it
24. If 250 have stored 1 might need it
25. If 2500 have stored 1 might need it

***Attitude (6-12)***

1. Would you like to store cord blood stem cells for this pregnancy?
2. Yes
3. No
4. Can’t say, as I don’t know much about it.
5. I am interested, but would first like to know more about it
6. If the answer of ‘Item 6’ is ‘Yes’. Where?
7. Public Bank
8. Private Bank
9. Don’t know the difference between A & B
10. If the answer of ‘Item 6’ is ‘Yes’. Why?
11. I feel this is like a health insurance for my family
12. I feel this is a unique opportunity, I don’t want to miss
13. It might help my family sometime
14. If Answer of Item 6 is ‘No’. Why?
15. Inadequate Information
16. Not affordable
17. Family pressure/problems
18. Don’t know how to go about it
19. Would you like to bank cord blood banking for research (if given an option), keeping in mind the larger picture of future benefit to the society
20. Yes, in any case
21. Yes if there is some financial compensation to me
22. Yes if it is totally harmless to me and my baby
23. Never
24. Which source do you rely upon to gather information related to the issues of cord blood banking?
25. Your obstetrician
26. Cord blood bank representative
27. Social networks (Facebook/Twitter)
28. Google
29. Family/Friends
30. Would you like to be informed about cord blood banking NOW?
31. Yes
32. No…..

Because of - a) Lack of time b) Not interested c) Don’t feel comfortable d) Other reason (please specify)

***Expectation (13-15)***

1. Upto how many years you think cord blood stem cells can be stored? (approx.)
2. 10 years
3. 20 years
4. 30 years
5. 40 years
6. What do you feel stem cells can be used in future for?
7. Treating systemic diseases (like high blood pressure, diabetes)
8. Cancer treatment
9. Treating systemic diseases and cancer
10. Regenerating new organs
11. Treating systemic diseases, cancers and regenerating organs
12. What is the source of your knowledge regarding this issue of cord blood banking?
13. Internet
14. Television
15. Family/Friend
16. Doctor
17. Cord blood bank representative
18. Others (please specify):
